# Clarifying the synergistic effects of emotion dysregulation and inhibitory control on physical aggression

**DOI:** 10.1002/hbm.26012

**Published:** 2022-07-15

**Authors:** Nadia Bounoua, Jeffrey M. Spielberg, Naomi Sadeh

**Affiliations:** ^1^ Department of Psychological & Brain Sciences University of Delaware Newark Delaware USA

**Keywords:** cognitive control, cortical thickness, emotion–cognition interactions, violence perpetration

## Abstract

Rising rates of violence underscore the need to better understand how systems that regulate distress and impulse control jointly modulate aggression risk. The goals of the current study were to investigate the unique and interactive effects of emotional dysregulation and inhibitory control on the perpetration of physical aggression. We recruited a high‐risk community sample of 206 adults (M/SD_age_ = 33.55/10.89 years old; 47.1% female) who reported a range of physically aggressive behaviors. All participants completed a self‐report measure (Difficulties in Emotion Regulation Scale), neuropsychological testing (Color Word Interference Test), and clinical interviewing (Lifetime History of Aggression Interview), and a subset of individuals (*n* = 134) underwent a neuroanatomical scan. As expected, the interplay of emotional and inhibitory control explained unique variance in physical aggression above and beyond their main effects. The positive association between emotion dysregulation and aggression strengthened as inhibitory control decreased. Cortical thickness in two right prefrontal clusters, one that peaked in the superior frontal gyrus and one that peaked in the caudal middle frontal gyrus, was also associated with the interactive effects of emotional dysregulation and inhibitory control. Notably, thickness in the superior frontal gyrus mediated the association between emotion dysregulation and physical aggression at low levels of inhibitory control. Using a multilevel and multimethod approach, the present study revealed neuroanatomical correlates of emotion–cognition interactions that have translational relevance to violence perpetration. These findings extend previous work primarily focused on functional‐based neural assessments and point to the utility of examining neuroanatomical correlates of emotion–cognition interactions for understanding human aggression.

## Graphical abstract

This study found that cortical thickness in the right superior and caudal middle frontal gyrus was associated with the interaction between emotion regulation and inhibitory control. Further, thickness in this region accounted for frequency of engagement in violent behaviors. Together, findings from this study suggest that this neural cluster may represent a neurobiological instantiation of emotion–cognition interactions that has translational relevance to violence perpetration.

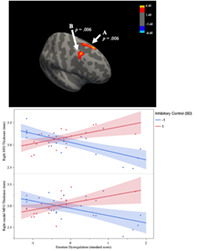

## 1 INTRODUCTION

According to the Center for Disease Control, the perpetration of violence is a leading cause of morbidity and mortality in the United States. Research suggests that there are individual differences in the capacity to manage the downstream effects of stress on emotional and cognitive systems that modulate violence risk. Emotion regulation, for example, is a set of processes that is key for managing the negative affective states that arise in stressful situations, with research suggesting individuals prone to emotional dysregulation (i.e., lower emotion regulation capacity) are at elevated risk for aggressive behavior and worse psychosocial outcomes (for review, see Roberton et al., 2012). Similarly, deficits in inhibitory control, a process that is crucial for regulating behavioral urges, have been linked to an array of harmful behaviors, including aggression (Hofmann et al., 2012). Despite their relevance for explaining aggression, relatively few studies have empirically investigated the interplay between emotion dysregulation and cognitive control on violence perpetration, and no studies have investigated the neuroanatomical substrates associated with these synergistic effects. To address these gaps, the present study examined the unique and interactive effects of emotional dysregulation and inhibitory control—across behavioral and morphological levels of analysis—on aggression.

### 1.1 Disruptions in emotion regulation and cognitive control as determinants of aggression

Emotion dysregulation, or the inability to effectively manage difficult emotions (Gratz & Roemer, 2004; Gross, 1998), has been identified as a key risk factor for engagement in a range of impulsive and self‐destructive behaviors, including the perpetration of violence against others (Garofalo et al., 2018; Garofalo & Velotti, 2017; Miles et al., 2017; Newhill et al., 2012; Sullivan et al., 2010). For example, difficulties with emotion regulation have been shown to mediate the association between personality disorder symptoms and aggressive acts (Domes et al., 2006; Garofalo et al., 2020; Scott et al., 2014). Additionally, research shows that individuals who struggle to manage strong negative emotions, like anger, may engage in aggressive acts as a maladaptive coping strategy to relieve negative affect (Bushman et al., 2001; Gardner & Moore, 2008; Roberton et al., 2012; Sullivan et al., 2010).

Despite robust associations between emotion dysregulation and aggression, not everyone who experiences difficulty regulating negative emotions goes on to perpetrate violence against others, indicating that additional regulatory mechanisms are important to consider in etiological models of aggression. There is considerable evidence to suggest that emotional processing is reciprocally interconnected with, and influenced by, cognitive functions (Blair et al., 2007; Pessoa et al., 2002), which are also integral for self‐control (Nigg, 2000). Inhibitory control is a higher‐order executive function that aids the ability to inhibit prepotent responses or impulses in service of goal‐directed behaviors (Diamond, 2013; Friedman & Miyake, 2004; Nigg, 2000), with research showing that deficits in inhibitory control confer risk for aggression (Fatima & Sharif, 2017; Hancock et al., 2010; Pawliczek et al., 2013). Deficits in inhibitory control may be exacerbated in individuals high on emotional dysregulation, given evidence that strong emotional states can compromise the ability to effectively engage the inhibitory control processes necessary for regulating impulsive behaviors (Verona et al., 2012; Verona & Kilmer, 2007). For example, research shows that individuals with personality disorders characterized by heightened reactivity to emotional stimuli, like antisocial and borderline personality disorders, demonstrate disruptions in executive control processes in negative emotional contexts (Sprague & Verona, 2010; Verona et al., 2012). These findings are consistent with evidence that deficits in executive functioning are a key contributor to stress‐induced aggression broadly (Sprague et al., 2011), with one experimental study showing that individuals with low trait self‐control are more likely to engage in aggressive acts following provocation than those individuals with greater trait self‐control (DeWall et al., 2007).

Based on this body of research, it is clear that the capacity to refrain from aggressive behavior depends on both intact emotional and cognitive processing, which coincides with theories of aggression that implicate the dynamic interplay of emotion regulation and inhibitory control (Anderson et al., 1996; Anderson & Bushman, 2002; Finkel, 2014). Notably, some empirical studies have shown that the interaction of emotion regulation and inhibitory control processes provides incremental validity in predicting aggression over these processes in isolation (Holley et al., 2017; Hsieh & Chen, 2017). These findings suggest that the extent to which individuals are able to effectively engage inhibitory control and emotion regulation systems are intertwined and should be considered in models of aggressive behavior. However, the extent to which these cognition–emotion interactions are instantiated at a neural level is less understood. Identifying neurobiological substrates in emotion regulation and inhibitory control might help to identify individuals at greatest risk for engaging in violent behaviors.

### 1.2 Neuroimaging studies of aggression and emotion–cognition interactions

Previous research on the neurobiology of aggression implicates deficient top‐down control systems in prefrontal structures responsible for regulation of affective processes and behavioral urges as key neural mechanisms of aggression (Siever, 2008). Unsurprisingly, less cortical thickness and volume in the prefrontal cortex (PFC), which contains key regions relevant to executive function and regulation of sensory and emotionally salient information, has been associated with an increased tendency to engage in violent acts (Blair, 2016; Chester et al., 2017; Hofhansel et al., 2020; Ling et al., 2019; Yang & Raine, 2009). For example, one study showed that thinner cortex in PFC mediated the association between childhood trauma exposure and lifetime aggression in a sample of community adults (Bounoua et al., 2020). Together, these results support the notion that morphometry of the PFC is associated with aggressive tendencies.

Given that engagement in aggressive acts tends to be influenced by both emotional states and one's ability to modulate and inhibit behavioral responses, it follows that the neural substrates of both emotional processing and inhibitory control need to be considered in etiological models of aggression (Bertsch et al., 2020; Blair, 2016; Pessoa, 2008). Indeed, previous neuroimaging work has shown that these neural systems are mutually influential during emotional challenges (Bartholomew et al., 2019; Kaiser et al., 2015; Lindquist et al., 2012; Okon‐Singer et al., 2015). For example, one study found that disrupted connectivity in neural networks that support both emotion regulation and impulse control was associated with increased risk for externalizing behaviors (Sadeh et al., 2019). Taken together, this work highlights the importance of future research to examine the interplay of inhibitory control and emotion regulation processes at a neural level (Bartholomew et al., 2021). However, the majority of this research has utilized functional MRI studies to examine these emotion–cognition interactions, with relatively less work focusing on neuroanatomical substrates that support these processes. Further, no studies, to our knowledge, have examined the cortical substrates associated with the interactive relationships between emotion dysregulation and deficits in inhibitory control, and how these relate to aggressive behavior.

### 1.3 Current study

While emotion dysregulation has been identified as a clear risk factor for aggression, further research is needed to understand the degree to which individual differences in cognitive control attenuate or exacerbate or mitigate this issue. The goal of the current study was to build upon existing literature on aggression by examining the extent to which inhibitory control modulates the link between emotion dysregulation and lifetime engagement in aggressive behavior.

Hypothesis 1 *We predicted that emotion dysregulation would be positively associated with aggression and that inhibitory control would moderate this association, such that the association would be stronger in individuals with weaker inhibitory control*.

Hypothesis 2 *We explored whether inhibitory control moderated the association between emotion dysregulation and cortical thickness as a putative intermediate mechanism of the tendency to engage in aggression. Given the role of PFC in top‐down regulation, we predicted that emotional and cognitive dysregulation would be associated with thinner PFC cortex*.

Hypothesis 3 *We tested whether cortical thickness in identified clusters mediated the association between emotion dysregulation and lifetime physical aggression*.

## 2 METHODS

### 2.1 Participants

The sample consisted of 206 community adults (97 women [47.1%]) aged 18–55 (*M/SD* = 33.6/10.9) who were recruited using flyers and online advertisements for a study on risky behavior. Advertisements stated that interested individuals may be eligible to participate if they were between the ages of 18–55 and fluent in the English language. Individuals who reported serious medical or neurological conditions, or endorsed MRI contraindications were ineligible to participate, as data collection was part of a larger study on neural networks of inhibitory control. All participants with complete self‐report, interview, neuropsychological data were included in the analysis.

Approximately 62.5% of participants identified as White/Caucasian, 27.2% as Black/African‐American, 8.7% as Asian, and 3.4% as another race. Approximately 7.8% of the sample identified as Hispanic or Latinx. The majority of participants came from socioeconomically disadvantaged neighborhoods in Wilmington, Delaware (https://www.neighborhoodscout.com/de/wilmington/crime; Wilmington, DE Crime Rates, 2021). In our sample, past year household income ranged from $0 to >$100,000 a year, with the average reported income ranging from $25,000 through $49,999. The most common level of educational attainment was a high school diploma equivalent or less (52.4%), with the remainder of participants reporting an associate's degree (11.7%), bachelor's degree (20.9%), or graduate degree (15.0%). Slightly less than half of the sample (47.6%) reported prior justice‐system involvement in their lifetime. Approximately 58.1% of the sample reported some form of traumatic experience before the age of 13 years old. More than half (58.3%) of the sample met criteria for at least one externalizing disorder (alcohol use = 32.5%, substance use = 42.7%, gambling = 5.3%, antisocial personality = 9.2%), and 48.5% met criteria for at least one internalizing disorder (major depressive = 42.2%; generalized anxiety = 13.6%; panic = 2.4%; social anxiety = 8.3%).

A subset of individuals (*n* = 134) completed an MRI visit in addition to the initial assessment. We tested for differences between individuals with and without MRI data and found that the two groups did not differ in terms of lifetime aggression (*F*_(1,204)_ = 1.74, *p* = .19), emotion dysregulation (*F*_(1,204)_ = 2.77, *p* = .10), inhibitory control (*F*_(1,204)_ = 2.00, *p* = .16), age (*F*_(1,204)_ = 3.19, *p* = .08), income (*F*_(1,203)_ = 2.21, *p* = .14) or gender (*X* ^2^_(1,206)_ = 0.72, *p* = .40).

### 2.2 Measures

#### 2.2.1 Physical aggression

The Lifetime History of Aggression Interview (Coccaro et al., 1997) was administered by trained graduate students or a Clinical Psychologist to assess for lifetime engagement in physical aggression. For the current study, we used responses to three questions about the frequency of physical aggression since age 15: “How many physical fights have you been in?” “How many times have you assaulted another person?” and “How many times have you broken things when you were angry, like windows, dishes, or destroying property?” Consistent with the validation paper (Coccaro et al., 1997), each item was rated on the following scale: no events (0), one event (1), a couple or few events (2), several or some events (3), many events (4), or so many events that they cannot be counted (5). A total aggression score (range: 0–4.67; *M/SD* = 1.07/1.21) was created by taking the average of the three items (Cronbach's alpha = .70).

#### 2.2.2 Emotion dysregulation

The Difficulties in Emotion Regulation Scale, Brief Version (DERS‐16) (Bjureberg et al., 2015) is a well‐validated 16‐item self‐report measure of emotional regulation. Sample items include “When I'm upset, I feel out of control” and “When I'm upset, my emotions feel overwhelming.” Participants were asked to respond to each item on a Likert scale ranging from “Almost never” (0) to “Almost always” (5). A total emotion dysregulation score was calculated by averaging the items (range = 1.00–4.94; *M/SD* = 2.01/0.88), with higher scores indicating greater emotion dysregulation (Cronbach's alpha = .96).

#### 2.2.3 Inhibitory control

The Color‐Word Interference Test (CWIT), a neuropsychological test from the Delis‐Kaplan Executive Function System (D‐KEFS; Delis et al., 2001) was used to measure inhibitory control. On the inhibitory control subtest, participants were presented with a list of color words and asked to name the color of the ink in which each word was printed as quickly as possible without making mistakes. Similar to the classic Stroop (1935) test, participants were required to inhibit the prepotent response to read the color word (task‐irrelevant) when naming the ink color for each word. Scaled scores of completion time from this subtest were created to provide an index of inhibitory control (range: 1–16; *M/SD* = 10.75/2.71). A continuous measure of inhibitory control was used in all analyses.

#### 2.2.4 Cortical thickness

A T1‐weighted multiecho (ME) MPRAGE (resolution = 1 mm^3^, TR = 2530 ms, TEs = 1.69, 3.55, 5.41, 7.27 ms) was collected was collected on a Siemens 3 T Magnetom Prisma scanner with a 64‐channel head coil. The MEMPRAGE has the advantage of less distortion and higher gray/white contrast than standard MPRAGE sequences, resulting in more reliable cortical models (van der Kouwe et al., 2008). A T2‐weighted variable flip‐angle turbo spin‐echo scan (resolution = 1 mm^3^, TR = 3200 ms, TE = 564 ms) was collected, which is used in FreeSurfer to better differentiate the gray‐matter/dura boundary. The thickness of the cortical mantle at each vertex was estimated using FreeSurfer's (v6) standard morphometric pipeline (Salat et al., 2004). Quality assurance was conducted using procedures similar to the steps outlined in the Qoala‐T manual (Klapwijk et al., 2019). First, both T1 and T2 images were visually inspected for artifacts, motion, and so on. Following FreeSurfer's standard pipeline, at least two trained raters examined the data for errors, including the inclusion of dura or skull after brain extraction or errors in the pial or white matter surfaces. Data were spatially smoothed using a Gaussian kernel of 10‐mm full‐width at half maximum.

### 2.3 Procedures

Written and oral consent was obtained from all individuals prior to participation. All participants completed a battery of self‐report measures, a clinical interview, and neuropsychological testing. A subset of individuals also completed a neuroimaging protocol. The University Institutional Review Board approved all protocols and procedures (Protocol #: 1361164). Clinical interviews and neuropsychological tests were conducted by graduate students and research staff who were trained by a licensed clinical psychologist. Each assessor completed didactic sessions and were observed until reliability with the supervisor was reached. Weekly group supervision was held to review assessments as they were completed.

### 2.4 Data analytic plan

Bivariate correlations were conducted to assess the associations between key study variables. Next, a moderation analysis was conducted to examine whether inhibitory control moderated the association between emotion dysregulation and lifetime engagement in physical aggression (*Hypothesis* *1*). Moderation analyses were conducted using Model 1 in PROCESS Macro v3.3 (Hayes, 2018).

FreeSurfer's QDEC was used to test for the main and interactive effects of emotion dysregulation and inhibitory control on cortical thickness using general linear models (*Hypothesis* *2*). Multiple comparisons were corrected for using FreeSurfer's standard Gaussian Monte Carlo procedure (10,000 iterations) with a vertex‐wise threshold of *p* < .001 and a cluster‐wise threshold of *p* < .05 (Greve & Fischl, 2018; Hagler et al., 2006), taking into account both hemispheres. Explanatory and dependent variables were examined for outliers and nonlinear distributions.

Finally, a moderated‐mediation analysis was conducted using Model 8 in PROCESS Macro v3.3 (Hayes, 2018). This analysis was used to test our hypothesis that the relationship between emotion dysregulation and physical aggression was mediated through cortical thickness as a function of inhibitory control (*Hypothesis* *3*).

Age, biological sex, educational attainment, and body mass index were entered as covariates of no interest in all subsequent analyses, given associations between these variables and neural morphology in previous research (Chen et al., 2007; Lavagnino et al., 2016). Effect sizes were determined using standardized coefficients as appropriate.

### 2.5 Transparency and openness

We report how we recruited our sample, all data inclusion and exclusion criteria, and all measures in the study, and we follow JARS (Kazak, 2018). Data were analyzed using the PROCESS Macro v3.3 (Hayes, 2018) in SPSS v28 (IBM Corp, 2021) and FreeSurfer's QDEC application. This study's design and its analysis were not preregistered. A subset of the data used in this study have been uploaded to the NIMH Data Archive. All raw data used in this study can be made available by contacting the corresponding author.

## 3 RESULTS

### 3.1 Descriptive statistics and bivariate correlations

Approximately two thirds of the sample (66.5%) reported engaging in at least one physically aggressive act and 38.1% reported two or more physically aggressive acts. At a bivariate level, emotion dysregulation was positively correlated with a lifetime history of physical aggression (*r* = .23, *p* < .001), replicating previous findings linking emotional dysregulation with the likelihood of engagement in physical altercations. Similarly, inhibitory control was also associated with physical aggression (*r* = −.16, *p* = .02), indicating that engagement in physical aggression was associated with less ability to inhibit automatic responses. Interestingly, emotion dysregulation and inhibitory control were not correlated in our sample (*r* = −.01, *p* = .90), suggesting that these represent distinct constructs relevant to understanding aggression. Age (*r* = .28, *p* < .001) and gender (*F*_(1,204)_ = 9.96, *p* = .002) were significantly associated with aggression, such that older individuals and men reported more aggressive behaviors than younger adults and women.

### 3.2 Inhibitory control moderates the link between emotional dysregulation and aggression

**FIGURE 1:**
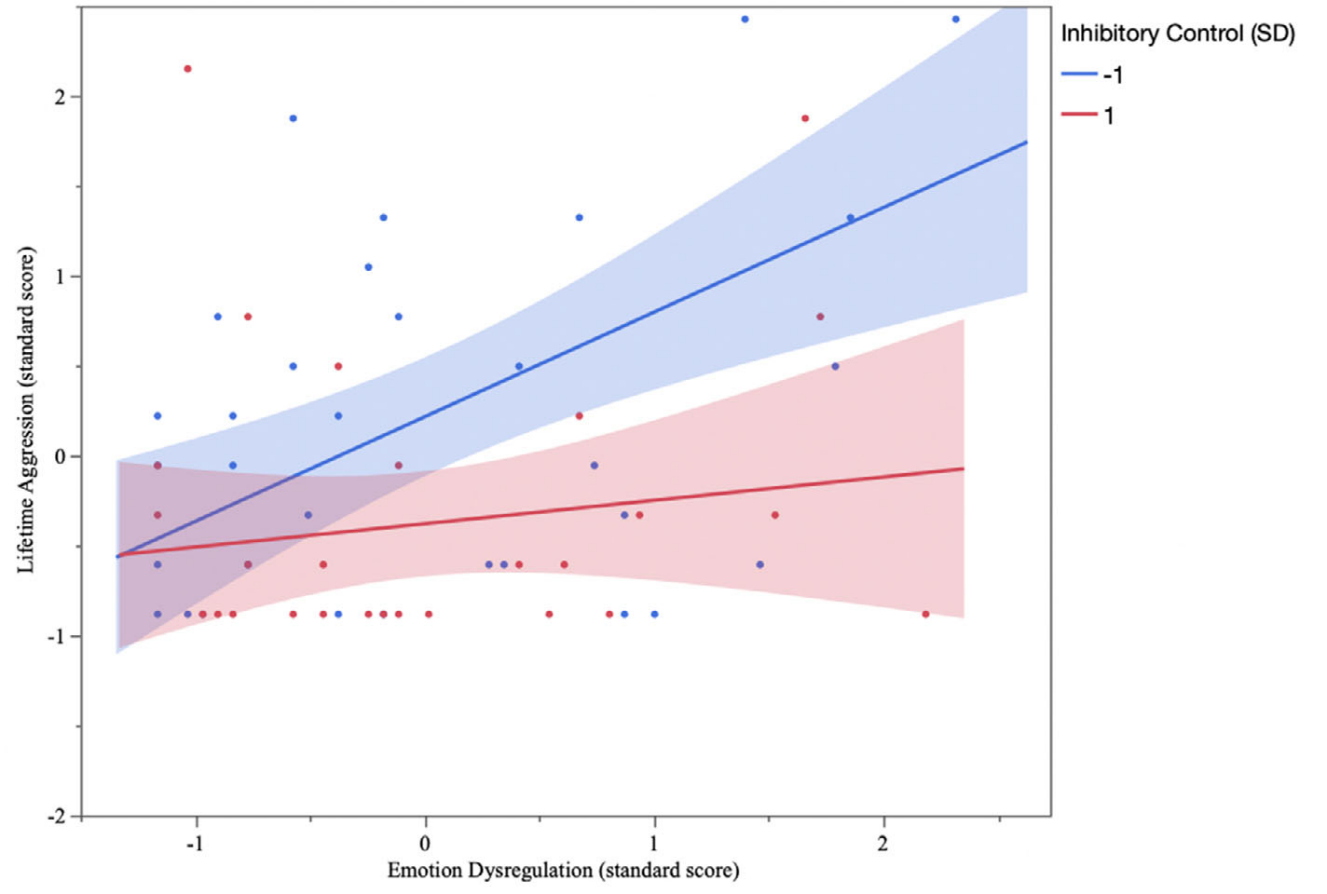
Inhibitory control moderates the association between emotion dysregulation and aggression perpetration.

We next examined the main and interactive effects of emotion dysregulation and inhibitory control on lifetime aggression. Age (*t* = 4.62, *p* < .001) and biological sex (*t* = 3.31, *p* = .001) remained significant predictors of aggression. Emotion dysregulation remained positively related to lifetime aggression when other predictors were included in the model (*t* = 4.53, *p* < .001), but inhibitory control did not (*t* = −1.49, *p* = .14). As hypothesized, the emotion dysregulation × inhibitory control interaction was significant, indicating that the association between emotion dysregulation and physical aggression was moderated by inhibitory control (*F*_(1,200)_ = 4.74, *p* = .03, 95%CI [−0.13, −0.01], see Figure 1). Specifically, at low levels of inhibitory control (−1 SD), emotion dysregulation was associated with more engagement in physically aggressive acts (*t* = 4.76, *p* < .001, 95%CI [0.32, 0.77]), whereas it was not positively related to aggression at high levels (+1 SD) of inhibitory control (*t* = 1.42, *p* = .16, 95%CI [−0.06, 0.41]).

### 3.3 Cortical thickness associations with emotion dysregulation and inhibitory control

**FIGURE 2:**
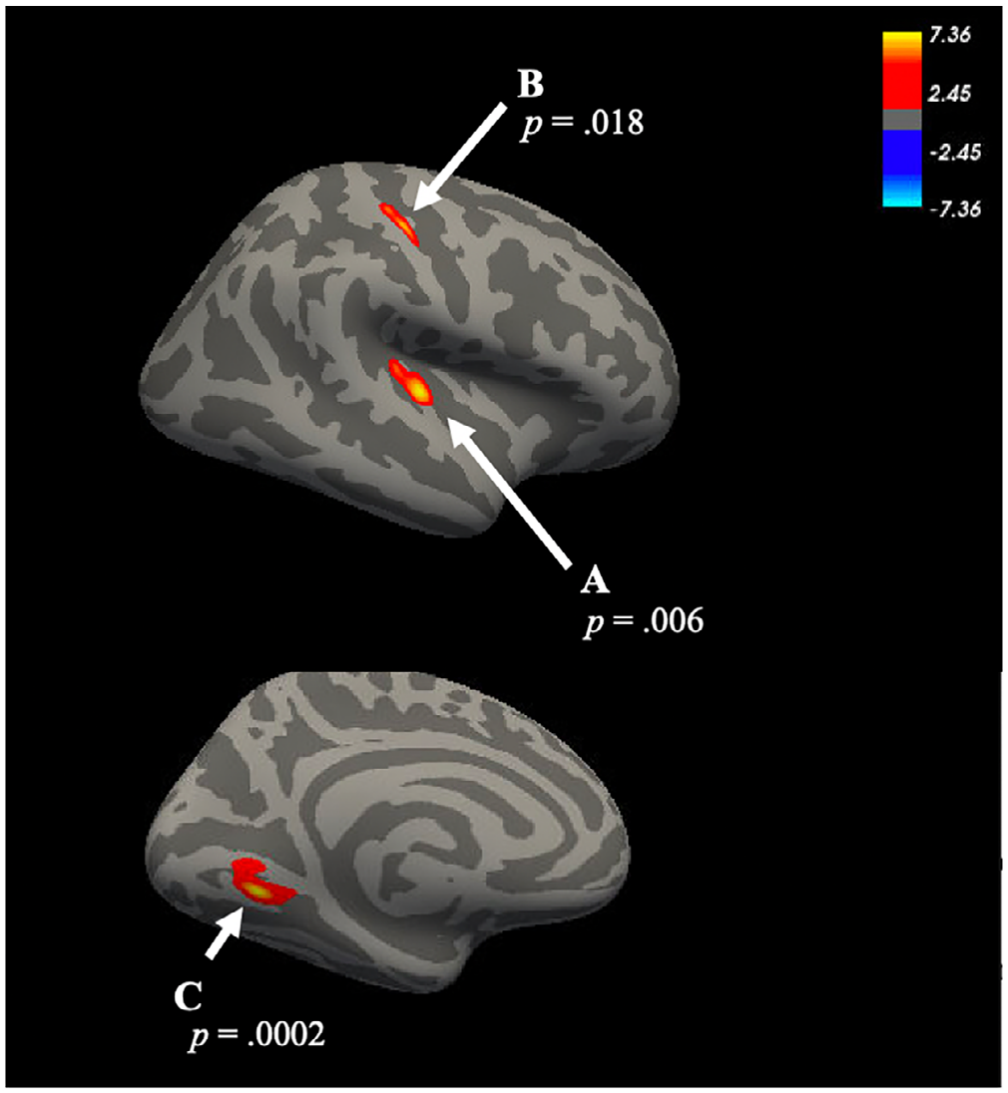
Inhibitory control is associated with cortical thickness in the (a) right transverse temporal gyrus, (b) right postcentral gyrus, and (c) left lingual gyrus

There was a main effect of inhibitory control on three clusters, such that higher inhibitory control was associated with thicker cortex (see Table 1 and Figure 2). The first cluster was located in right transverse temporal gyrus (*cluster‐wise p value* = .006). The second cluster peaked in the right postcentral gyrus (*cluster‐wise p value* = .018). The third cluster was located in the left lingual gyrus (*cluster‐wise p value* = .0002). Follow‐up tests indicated that lifetime physical aggression was not associated with cortical thickness in any of these clusters (all *p*'s > .27). There was no main effect of emotion dysregulation on cortical thickness.

**TABLE 1:** Main and interactive effects of emotion dysregulation and inhibitory control on cortical thickness

Hemisphere	Annotation	Peak *F*‐value	Peak MNI (*x*, *y*, *z*)	No. of vertices	Cluster size (mm^2^)
Main effect of inhibitory control					
Right	Transverse temporal	4.298	44.7, −21.0, 5.5	778	294.42
Right	Postcentral	3.995	46.0, −17.3, 50.8	451	246.19
Left	Lingual	7.360	−19.0, −64.8, −6.8	783	487.21
Interaction effect of emotion dysregulation × inhibitory control					
Right	Superior frontal	4.447	15.7, 30.2, 52.3	504	295.65
Right	Caudal middle frontal	4.146	35.2, 14.3, 51.2	436	295.33

*Note*: *N =* 134. All clusters survived Monte Carlo Simulation correction for multiple comparisons (*p* < .001) across both hemispheres (Bonferroni = 2). Annotations generated using Desikan–Killiany Atlas (Desikan et al., 2006). There were no main effects of emotion dysregulation on cortical thickness.

**FIGURE 3:**
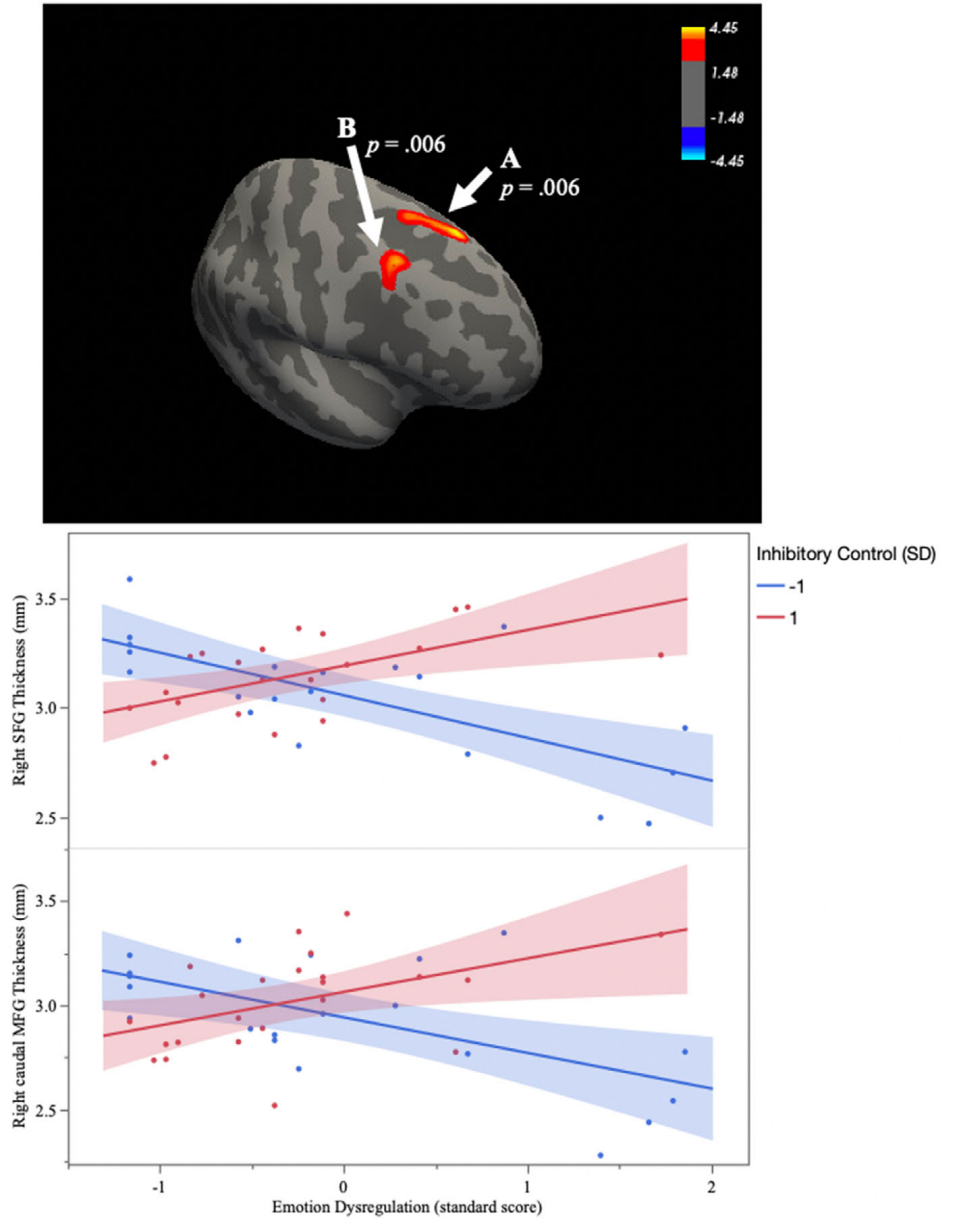
Emotion dysregulation differentially relates to thickness in two clusters in the right prefrontal cortex as a function of inhibitory control: (a) superior frontal gyrus and (b) caudal middle frontal gyrus

Two clusters in the right hemisphere emerged in which emotion dysregulation and inhibitory control interacted to predict cortical thickness. The first cluster was located in the superior frontal gyrus (SFG; *cluster‐wise p value* = .006) and the second cluster was in the caudal middle frontal gyrus (caudal MFG; *cluster‐wise p value* = .006) (see Table 1 and Figure 3). Post hoc probing of the interaction revealed that, at low levels (−1 SD) of inhibitory control, emotion dysregulation was associated with thinner cortex, whereas this association became nonsignificant at high levels (+1 SD) of inhibitory control. Follow‐up analyses revealed that cortical thickness in both the SFG cluster (*partial r* = −.29, *p* = .001) and the caudal MFG (*partial r* = −.18, *p* = .038) was negatively associated with lifetime engagement in physical aggression.

### 3.4 Inhibitory control moderates the indirect effect of emotion dysregulation on aggression through cortical thickness

**FIGURE 4:**
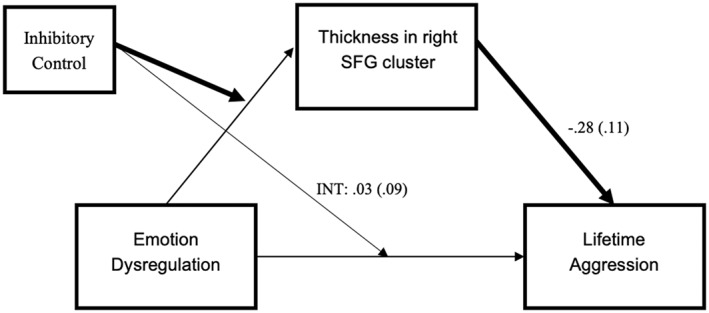
Inhibitory control moderates the indirect effect of emotion dysregulation and physical aggression via cortical thickness in the right superior frontal gyrus. Standardized beta coefficients and standard errors are represented. Bolded lines represent significant pathways. INT refers to the interaction effect between emotion dysregulation and inhibitory control.

We conducted a moderated‐mediation analysis to examine the conditional indirect effect of emotion dysregulation on lifetime physical aggression via cortical thickness in the two frontal regions. The index of moderated‐mediation was significant for the indirect effect via right SFG thickness (95% CI [−0.21, −0.02]), indicating that cortical thickness in the SFG mediated the association between emotion dysregulation and lifetime aggression, but only among individuals with low levels of inhibitory control (*DKEFS* scaled scores <9) (see Figure 4). Examination of the individual paths indicated that the interactive effect of emotion dysregulation and inhibitory control on cortical thickness was significant (conditional *a* path; *B* = 0.36, *p* = .0001, 95% CI [0.18, 0.53]), indicating that greater emotion dysregulation was associated with less SFG cortical thickness at low levels of inhibitory control. This effect was expected, given that this is how the cluster was identified. The direct effect of SFG thickness on physical aggression also remained significant (*b* path; *B* = −0.28, *p* = .0116, 95% CI [−0.50, −0.06]). Finally, the conditional direct effect of emotion dysregulation on physical aggression became nonsignificant when SFG cortical thickness was included in the model (*c*' path; *B* = 0.03, *p* = .772, 95% CI [−0.16, 0.21]). In total, the mediation analysis accounted for 36.25% of the variance on physical aggression (*p* < .001) and 41.55% of the variance in SFG cortical thickness (*p* < .001). Conversely, results showed that thickness in the caudal MFG did not mediate the association between emotion dysregulation and lifetime physical aggression (95% CI [−0.08, 0.09]).

#### 3.4.1 Supplementary analysis

We conducted supplementary analysis to test whether findings persisted after controlling for psychiatric disorders and childhood abuse exposure. The presence of a clinical disorder diagnosis was associated with greater aggressive behaviors (*r* = .41, *p* < .001) and emotion dysregulation (*r* = .35, *p* < .001), but not inhibitory control (*r* = −.05, *p* = .46). Childhood trauma exposure was associated with aggression (*r* = .31, *p* < .001), inhibitory control (*r* = −.20, *p* = .006), and clinical diagnosis (*r* = .26, *p* < .001), but not emotion dysregulation (*r* = .06, *p* = .38). Finally, we examined whether the moderated‐mediation remained significant after accounting for these variables, and results showed that the conditional indirect effect via SFG thickness remained significant after controlling for psychiatric history (95% CI = −0.126/−0.002) and childhood trauma (95% CI = −0.134/−0.002).

Next, we tested whether the interactive effect of emotion dysregulation and inhibitory control on cortical thickness in the identified regions differed between individuals with (*n* = 137) and without (*n* = 69) a history of aggressive behavior. Results showed the effects did not differ between the two groups (*p's* > .49).

## 4 DISCUSSION

The goal of this study was to examine the unique and interactive effects of emotion dysregulation and inhibitory control on engagement in physical aggression. We found that the link between emotional dysregulation and physical aggression became stronger at weaker levels of inhibitory control. Notably, the synergistic effects of emotional and cognitive dysregulation were also evident at a neuroanatomical level. PFC thickness, specifically in clusters in the SFG and caudal MFG, was associated with the interactive effects of emotional dysregulation and inhibitory control, and thickness in these regions was associated with lifetime aggression. Most notably, cortical thickness in the SFG region mediated the association between emotion dysregulation and physical aggression at low levels of inhibitory control, supporting the translational implications of these findings. Taken together, this study advances prior work by showing that the interaction of emotion dysregulation and inhibitory control is important for identifying neuroanatomical mechanisms supporting aggression perpetration.

### 4.1 Neuroanatomical markers of emotion–cognition dysregulation

Consistent with previous literature (Holley et al., 2017; Hsieh & Chen, 2017), we found that the risk conferred by emotional dysregulation for physical aggression was modulated by performance on an inhibitory control task. Specifically, emotion dysregulation evidenced a stronger positive link with aggression at low levels of inhibitory control, suggesting that individuals who experience difficulty with both emotional and inhibitory control are at greatest risk for aggression. In addition, the interactive effect of emotional dysregulation and inhibitory control was linked to cortical thickness in two clusters in the right SFG and caudal MFG cluster. Specifically, at low levels of inhibitory control, greater emotion dysregulation was associated with less cortical thickness in this cluster, whereas this effect was no longer significant at high levels of inhibitory control.

Structural variation in these regions has been linked to various regulatory systems, including inhibitory control of behavioral responses (Bertsch et al., 2020) and attentional control (Japee et al., 2015). This is consistent with functional‐MRI studies showing that neural activity in these regions is associated with control of impulsive responses (Hallowell et al., 2019; Hu et al., 2016). Further, previous work has also shown that SFG/MFG is highly interconnected with other structures involved in various regulatory systems, including motor and cognitive control networks and the default mode network (Briggs et al., 2020; Buckner et al., 2008; Li et al., 2013; Martino et al., 2011). Previous work using the same measure of inhibitory control (i.e., CWIT) has found significant links between right MFG structure and inhibitory control in a sample of healthy adults (Adólfsdóttir et al., 2014). In our sample, SFG and caudal MFG thickness was associated with inhibitory control, but only at high levels of emotion dysregulation. More studies will be needed to further elucidate the neural correlates of the interactive effects of emotion dysregulation and cognitive control, which may represent neuroanatomical markers of risk for behavioral dyscontrol and potential targets for treatment (Bartholomew et al., 2021; Milham et al., 2003; Siever, 2008).

Inhibitory control alone was positively associated with cortical thickness in three clusters that spanned the temporal, parietal, and occipital lobes. These neural regions have been implicated in various visuospatial and cognitive processes, including visual attention (e.g., Culham & Kanwisher, 2001; Freedman & Assad, 2009), verbal working memory (e.g., Jonides et al., 1998), and response inhibitory control (e.g., Garavan et al., 1999). Given these roles, our finding suggests that reduced cortical thickness in these clusters may reflect deficits in visual and cognitive processes involved in completion of the inhibitory control task.

### 4.2 Cortical thickness as an intermediate mechanism linking emotion dysregulation and aggression

We found that cortical thickness in the right SFG and caudal MFG cluster identified above was also associated with lifetime frequency of aggressive behavior. Specifically, thinner cortex in these clusters was associated with more physical aggression, mirroring findings from the Human Connectome Project (Zhu et al., 2020) and smaller studies with community adults (Sheehan et al., 2021) and violent offenders (Hofhansel et al., 2020). In combination with our finding that lifetime aggressive behavior was also predicted by the interaction between emotion dysregulation and inhibitory control, this finding suggests that thickness in this cluster may serve as an intermediate mechanism linking these psychological processes to aggression. To test this hypothesis, we conducted a moderated‐mediation model to test whether SFG and caudal MFG thickness explained the association between emotion dysregulation and physical aggression at low levels of inhibitory control. This hypothesis was partially supported by our finding of a significant conditional indirect effect via SFG (but not caudal MFG), which adds to the growing literature on etiological models of aggression by revealing that the extent to which emotion dysregulation relates to engagement in aggressive behaviors via neuroanatomical markers depends on cognitive functions. In other words, emotion dysregulation may be a robust determinant of aggression, but only when these are deficits in inhibitory control abilities. Further, cortical thickness in the right SFG cluster identified herein may serve as an intermediate mechanism linking emotional dysregulation and inhibitory control to behavioral dyscontrol (e.g., aggression). Future research will be needed to replicate and extend on the current findings by investigating the neurobiological instantiation of other risk mechanisms that lead to violence perpetration.

### 4.3 Strengths, limitations, and future directions

To our knowledge, this is the first study to provide empirical evidence of the neuroanatomical underpinnings of an emotion–cognition interaction in relation to aggression, complementing the existing work using functional‐based MRI studies. Among the study strengths is the multimodal approach, which allowed for the integration of multiple methods (i.e., self‐report, neuropsychological testing, clinical interviewing, structural MRI) to examine these processes across levels of analysis (i.e., behavioral, emotional, cognitive, neuroanatomical). Further, we sampled a relevant sample of community adults who were at high risk of violence exposure and perpetration, which extends previous work using convenience sampling or clinical samples of violent offenders.

However, there are some limitations, which restrict the interpretation of our findings. First, the cross‐sectional nature of our study inherently prevents our ability to make causal inferences about directionality of effects. For example, it may be the case that individuals with the tendency to engage in aggressive behaviors also have heritable vulnerabilities that influence cortical thickness and increase the likelihood for disinhibition and emotion dysregulation, though emotion dysregulation and inhibitory control were uncorrelated in this sample. Future longitudinal work will be needed to clarify the causal influences these constructs have on each other. Second, as with all measures that are self‐report in nature, the validity of participant self‐report responses is limited by a number of factors, including social desirability, lack of insight, and carelessness in responding. Given that these factors would only increase error and make it more difficult to detect consistent relationships among the study variables, we expect our present findings underestimate the strength of the observed relationships. Regardless, future research employing a multimethod assessment of emotion dysregulation is necessary to ascertain whether the current findings extend to more objective assessment measures. Third, our study was underpowered to investigate sex differences, and remains an area for future studies given existing work showing gender differences in regulatory processes and the tendency to engage in aggressive behaviors (Bennett et al., 2005; Koch et al., 2007; Rogier et al., 2019).

While the focus of the present study was on inhibitory control, existing research implicates other executive functions in relation to emotion dysregulation and aggression (Dutra & Sadeh, 2018; Pessoa et al., 2002). An interesting area for future research would be to investigate how different types of executive functions interact with emotion dysregulation to impact neuroanatomy and confer risk for violence perpetration. Further, while outside the scope of the present study, future studies should investigate the degree to which personality may influence the links between emotion–cognition interactions and aggression. Finally, given research linking childhood trauma to the perpetration of aggression toward others (Bounoua et al., 2020; Wright et al., 2019), future longitudinal research should examine how exposure to violence early in development may impact development of emotion regulation and inhibitory control processes relevant to aggression.

The present study extends previous research by providing new insight into the synergistic effects of emotional and cognitive dysregulation at behavioral and neuroanatomical levels of analysis. Further, it implicates the right superior frontal gyrus as a potential intermediate mechanism linking emotional and cognitive dysregulation with the perpetration of violence in adulthood. Results have the potential to inform etiological models of aggression and suggest targeting the perpetration of violence through the enhancement of inhibitory control may be beneficial at reducing aggression.

## CONFLICT OF INTEREST

The author declares that there is no conflict of interest.

## Data Availability

A subset of the data used in this study have been uploaded to the NIMH Data Archive. All raw data used in this study can be made available by contacting the corresponding author.
